# Temporal Sensitivity of Protein Kinase A Activation in Late-Phase Long Term Potentiation

**DOI:** 10.1371/journal.pcbi.1000691

**Published:** 2010-02-26

**Authors:** MyungSook Kim, Ted Huang, Ted Abel, Kim T. Blackwell

**Affiliations:** 1 George Mason University, The Krasnow Institute for Advanced Studies, Fairfax, Virginia, United States of America; 2 University of Pennsylvania, Department of Biology, Philadelphia, Pennsylvania, United States of America; University of Sussex, United Kingdom

## Abstract

Protein kinases play critical roles in learning and memory and in long term potentiation (LTP), a form of synaptic plasticity. The induction of late-phase LTP (L-LTP) in the CA1 region of the hippocampus requires several kinases, including CaMKII and PKA, which are activated by calcium-dependent signaling processes and other intracellular signaling pathways. The requirement for PKA is limited to L-LTP induced using spaced stimuli, but not massed stimuli. To investigate this temporal sensitivity of PKA, a computational biochemical model of L-LTP induction in CA1 pyramidal neurons was developed. The model describes the interactions of calcium and cAMP signaling pathways and is based on published biochemical measurements of two key synaptic signaling molecules, PKA and CaMKII. The model is stimulated using four 100 Hz tetani separated by 3 sec (massed) or 300 sec (spaced), identical to experimental L-LTP induction protocols. Simulations show that spaced stimulation activates more PKA than massed stimulation, and makes a key experimental prediction, that L-LTP is PKA-dependent for intervals larger than 60 sec. Experimental measurements of L-LTP demonstrate that intervals of 80 sec, but not 40 sec, produce PKA-dependent L-LTP, thereby confirming the model prediction. Examination of CaMKII reveals that its temporal sensitivity is opposite that of PKA, suggesting that PKA is required after spaced stimulation to compensate for a decrease in CaMKII. In addition to explaining the temporal sensitivity of PKA, these simulations suggest that the use of several kinases for memory storage allows each to respond optimally to different temporal patterns.

## Author Summary

The hippocampus is a part of the cerebral cortex intimately involved in learning and memory behavior. A common cellular model of learning is a long lasting form of long term potentiation (L-LTP) in the hippocampus, because it shares several characteristics with learning. For example, both learning and long term potentiation exhibit sensitivity to temporal patterns of synaptic inputs and share common intracellular events such as activation of specific intracellular signaling pathways. Therefore, understanding the pivotal molecules in the intracellular signaling pathways underlying temporal sensitivity of L-LTP in the hippocampus may illuminate mechanisms underlying learning. We developed a computational model to evaluate whether the signaling pathways leading to activation of the two critical enzymes: protein kinase A and calcium-calmodulin-dependent kinase II are sufficient to explain the experimentally observed temporal sensitivity. Indeed, the simulations demonstrate that these enzymes exhibit different temporal sensitivities, and make a key experimental prediction, that L-LTP is dependent on protein kinase A for intervals larger than 60 sec. Measurements of hippocampal L-LTP confirm this prediction, demonstrating the value of a systems biology approach to computational neuroscience.

## Introduction

Synaptic plasticity, the activity-dependent change in the strength of neuronal connections, is a cellular mechanism proposed to underlie memory storage. One type of synaptic plasticity is long term potentiation (LTP), which typically is induced by brief periods of high-frequency synaptic stimulation. LTP displays physiological properties suggestive of information storage and has been found in all excitatory pathways in the hippocampus, as well as other brain regions.

Late-phase LTP (L-LTP) is induced by 4 trains of stimulation separated by either 3–20 sec (massed) or 300–600 sec (spaced), lasts more than 3 hours, and requires protein synthesis [1]. Interestingly, the temporal spacing between successive trains regulates the PKA-dependence of L-LTP [2],[3]. A spaced protocol (using a 300 sec inter-train interval) requires PKA, whereas massed protocols (using 20 sec and 3 sec intervals) induce L-LTP that is independent of PKA. The mechanisms underlying this temporal sensitivity of PKA dependence are not understood.

PKA is composed of two regulatory subunits bound to two catalytic subunits that form a tetrameric holoenzyme. Sequential and co-operative binding of four cAMP to these regulatory subunits results in the release of two catalytic subunits [4],[5]. In the hippocampus, cAMP is produced by adenylyl cyclase types 1 and 8, which are activated by calcium and G_sα_ coupled receptors [6]. Consistent with this pathway of reactions leading to PKA, activation of dopaminergic and glutamatergic pathways is required for the induction of L-LTP in hippocampal CA1 pyramidal neurons [7]–[11]. NMDA receptor activation also leads to stimulation of the calcium sensitive isoform of adenylyl cyclase [12].

Because the induction of L-LTP involves complex networks of intracellular signaling pathways, computational models have been developed to gain an understanding of LTP [13]–[17]. Several of these studies, which specify the model using ordinary differential equations, explain the requirement for high frequency stimulation (e.g. 100 Hz for LTP) versus low frequency stimulation (e.g. 1 Hz for long term depression) in terms of the characteristics of CaMKII [18]–[21]. Even though PKA has been incorporated in some of these models, PKA activation is typically described using simplified algebraic equations [21]–[23]. These models do not include the role of dopamine or β-adrenergic receptors in PKA activation nor adequately describe the temporal dynamics of PKA activation. Consequently, these models do not evaluate the temporal sensitivity of PKA, and cannot accurately explain why PKA is required for spaced stimulation. In contrast, several models by Bhalla [24],[25] include not only the signaling pathways leading to PKA activation, but also those for mitogen activated protein kinase (MAPK) activation. However, Bhalla did not explore the role of dopamine or PKA in late-phase LTP, and we have utilized more recent experimental data to update several of the reactions, especially those involved in PKA activation.

To evaluate the biochemical mechanisms underlying the temporal sensitivity of PKA dependence of L-LTP and the role of dopamine, we developed a single compartment model of postsynaptic signaling pathways underlying L- LTP in CA1 pyramidal neurons of the hippocampus. Reaction rates and pathways are based on published biochemical measurements. Simulations explore the mechanisms underlying temporal sensitivity of LTP to PKA and complementary experiments test the model predictions of the critical temporal interval separating PKA-dependent and PKA-independent LTP.

## Results

**Figure 1:**
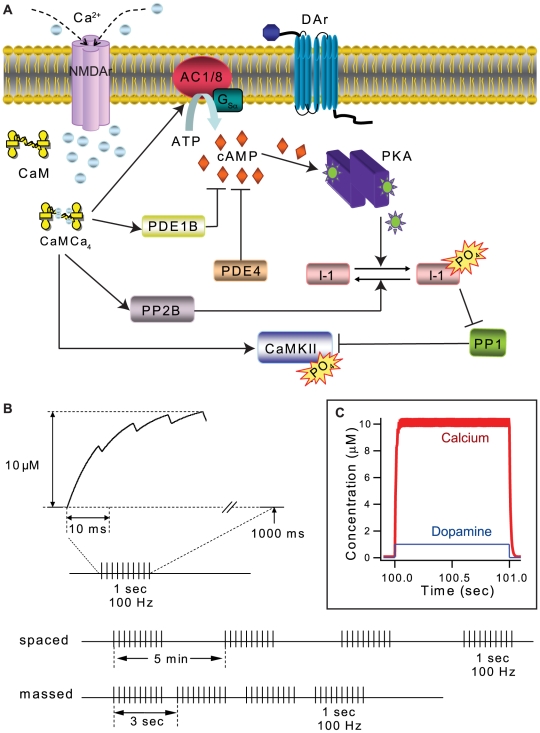
Model of L-LTP induction in hippocampal CA1 pyramidal neurons. (A) Signaling pathways in model activated by pre-synaptic stimulation. Calcium (from glutamate activation of NMDA receptors) binds to Calmodulin (CaM). Calcium-calmodulin can bind to and activate protein phosphatase 2B (PP2B), adenylyl cyclase types 1 and 8 (AC), phosphodiesterase type 1B (PDE1B), or CaMKII. Gs-coupled receptors, such as dopamine D1 and β-adrenergic receptors, synergistically activate adenylyl cyclase, which produces cAMP, which binds to PKA. Two types of phosphodiesterase (PDE1B and PDE4) degrade cAMP leading to PKA de-activation. Active PKA phosphorylates inhibitor-1 (I1), which then binds to and inhibits protein phosphatase 1 (PP1). CaMKII can autophosphorylate itself, and is dephosphorylated by PP1. (B) Stimulation protocols used for induction of L-LTP in the model. A single Ca^2+^ pulse rises instantaneously to an amplitude of 0.7 µM and decays with a time constant of 0.14 sec. Summation of Ca^2+^ pulses in the train gives a maximum elevation of Ca^2+^ of ∼10 µM (top panel). A spaced pattern consisted of four 100 Hz trains (each 1 sec duration and fixed total number of pulses (400)) with an inter-train interval of 300 sec. The massed pattern provides the same four 100 Hz trains, but with an inter-train interval of 3 sec. Time intervals shown are not to scale. (C) Simulated time course of Ca^2+^ and dopamine input for a single 1 sec train of synaptic input at 100 Hz with the magnitude of 10 µM and 1 µM respectively.

To evaluate the biochemical mechanisms underlying the temporal sensitivity of the PKA dependence of L-LTP, we developed a single compartment, computational model of signaling pathways underlying synaptic plasticity (Fig 1A and Tables 1 and 2). Elevations in intracellular calcium and dopamine binding to D1 type receptors activated signaling pathways leading to activation of CaMKII and PKA. Differential equations describing the biochemical reactions in the signaling pathways were numerically simulated using XPPAUT. As in experiments, the model was activated with four trains of 100 Hz stimuli using both spaced and massed inter-train intervals (Fig 1B). Each stimulus pulse in the 1 sec train produces a 7 msec influx in intracellular calcium, and each stimulus train produces a 1 sec elevation in extracellular dopamine (unless otherwise noted). Note that the total amount of imposed stimulation is identical for all cases – only the temporal pattern of stimulation is varied. In these simulations, enzyme activity is quantified as total (cumulative) activity over a simulated time period relative to the activity under conditions of no stimulation. For example, the cumulative activity for PKA is calculated as area under the curve describing concentration of the free catalytic subunit. Using cumulative activity over a simulated time is a better measure than at a single time point, such as the peak activity, because it represents the cumulative ability of the enzyme to phosphorylate downstream substrates such as AMPA receptors or inhibitor-1. For example, a kinase that has a high activity for 10 sec followed by a rapid decrease in activity phosphorylates fewer molecules than a kinase that has a high activity for 60 sec. Typically, biochemical experiments assessing kinase activity measure the change in concentration of a product [26],[27] after a specified duration, in recognition that the duration of kinase activity influences the total amount of phosphorylation measured.

**Table 1:** Reactions and rate constants of the dopamine activated pathway.

Reaction Equation	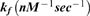	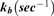	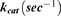
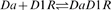	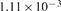	10	
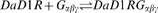	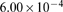	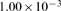	
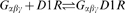	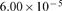	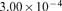	
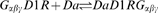	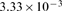	10	
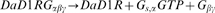	a20		
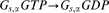	b10		
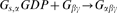	100		
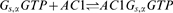	0.0385	10	
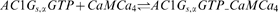	0.009	0.9	
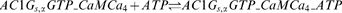	0.01	2273	
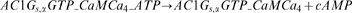			28.42
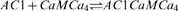	0.006	0.9	
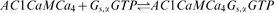	0.0385	10	
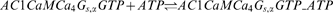	0.01	2273	
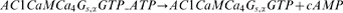			2.842
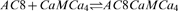	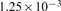	1	
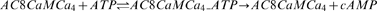	0.01	2273	2.842
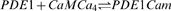	0.1	1	
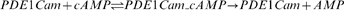	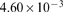	44	11
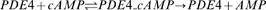	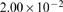	72	18
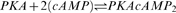	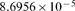	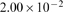	
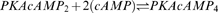	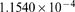	0.2	
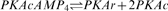	c 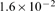	d 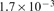	

a unit in *sec* ^-1^

b unit in *sec* ^-1^

c unit in *sec* ^-1^

d unit in *nM* ^-1^ *sec* ^-1^

Da, Dopamine; D1R, Dopamine receptor; G_αβγ_ , Heterotrimeric G protein; G_sα_GTP, Active stimulatory G_sα_ subunit bound to guanosine triphosphate) ; G_sα_GDP, Inactive G_sα_ subunit bound to guanosine diphosphate; G_βγ_ , Dimeric G protein subunits dissociated from the α subunit of the trimeric G protein; CaMCa_4_, the complex of four calcium ions bound to calmodulin; AC1, adenylyl cyclase type 1; AC8, adenylyl cyclase type 8; PDE1, Phosphodiesterase type 1B;

PDE1Cam, PDE1B bound with (and activated by) CaMCa_4_; PDE4, Phosphodiesterase type 4; PKA_r_, PKA regulatory subunit; PKA_c_, PKA catalytic subunit.

**Table 2:** Reactions and rate constants involved in signaling pathways from calcium to CaMKII and PP2B.

Reaction Equation	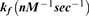	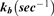	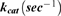
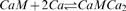	0.006	9.1	
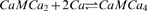	0.1	1000	
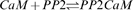	1	3	
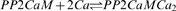	0.006	0.91	
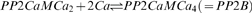	0.1	10	
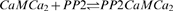	1	0.3	
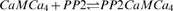	0.046	0.0012	
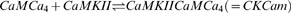	0.01	0.8	
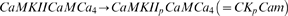	1 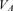		
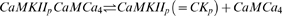	0.0008	0.0133	
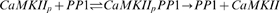	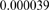	0.34	0.086
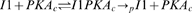	0.0014	5.6	1.4
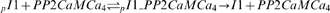	0.00467	11.2	2.8
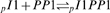	0.001	0.0011	
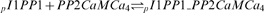	0.001	2	
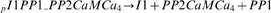			0.5

1 Unit in 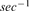.

CaM, calmodulin; PP_2_CaM, Protein Phosphatase 2B bound with apo-calmodulin; CKCam, CaMKII bound with CaMCa_4_; CK_p_Cam, CaMKII autophosphorylated at T^286^ with CaMCa_4_ trapped; CK_p,_ Autonomous CaMKII in which CaMCa_4_ dissociates but the subunit remains phosphorylated at T^286^; PP1, Protein Phosphatase 1; I1: inhibitor-1, pI1, Phosphorylated inhibitor-1.

### Simulated PKA activity is higher in response to spaced stimuli

**Figure 2:**
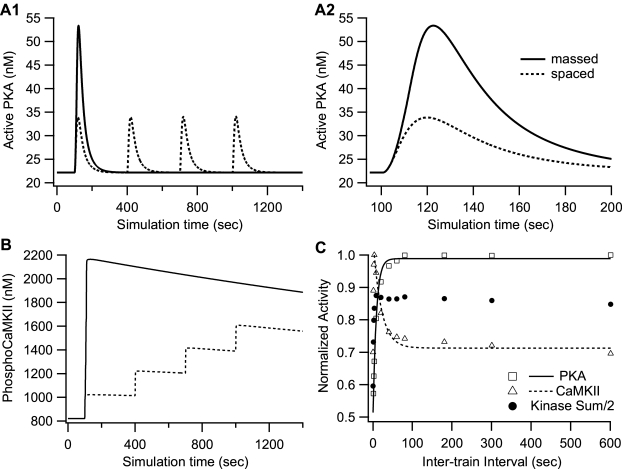
Temporal sensitivity of PKA and CaMKII activation during L-LTP induction. (A) Activation of PKA by different temporal patterns of stimulation. A1 shows 1400 sec of simulation time and A2 shows the first 200 sec to better show the four trains of massed stimulation. Though the peak is 1.4 times greater for massed trains, the cumulative activity of PKA activity is ∼60% greater for spaced trains (2321 nM-sec versus 1455 nM-sec). (B) The increase in phosphoCaMKII activity (sum of Ca_4_-calmodulin trapped and autonomous forms) is smaller for spaced than massed stimulations. The positive feedback loop is visible in the spaced case, in which increments of phosphorylated CaMKII increase with subsequent trains. (C) Cumulative PKA activity (measured as area under the curve) shows an exponential increase (τ = 8.5 sec) as inter-train interval is increased. The peak phosphoCaMKII decreases exponentially with temporal interval, exhibiting a frequency sensitivity opposite to that of PKA. The time constant of the decrease, τ, is 20.8 sec. The sum of the normalized kinase activity (divided by two for graphical purposes) is constant for inter-train intervals greater than 3 sec, suggesting that the increase in PKA is compensating for a phosphoCaMKII deficit with larger for inter-train intervals.

Simulation results show that the activation of PKA is greater with spaced as compared to massed stimulation (Fig 2A1). These results are consistent with experimental results [3] showing that PKA is required for spaced, but not massed stimulation. The cumulative activity of PKA with spaced stimulation (2321 nM-sec) is 60% greater than with massed stimulation (1455 nM-sec). Although the massed protocol produces a higher peak PKA activity, it is not 4 times higher than the peak produced from a single spaced train of stimulation because of sub-linear summation: the PKA peak activity for massed stimulation is only 1.4 times higher than the peak activity in response to spaced stimulation (Fig 2A2). Subsequent trains do not increase the peak activity of PKA, but do contribute to cumulative PKA activity over time by linear summation; therefore more PKA activity is available with spaced stimuli.

Simulations are repeated for a range of inter-train intervals to further explore the temporal sensitivity of PKA dependence. Fig 2C shows that cumulative PKA activity increases with temporal interval, with a time constant, τ,of 8.5 sec. PKA activity reaches 95% of maximal value within 3 time constant, i.e., at 25.5 sec. This temporal sensitivity is not observed if peak activity is evaluated. Activity at a single time point, such as 10 minutes after stimulation, is often used to compare with experimental measurements that measure enzyme activity at a single time point. Nonetheless, cumulative activity better indicates the ability of an enzyme to act on downstream targets. Using single time point measures of activity may explain why a previous study did not observe temporal sensitivity of PKA.

### Temporal sensitivity of CaMKII activity is opposite to that of PKA

This increase in PKA activity with increasing inter-train interval can partly explain the mechanism of temporal sensitivity of PKA dependence of L-LTP, but the other part of the explanation is likely a deficit in some other molecule, such as CaMKII, which is known to be sensitive to higher frequency stimuli and plays a major role in LTP. Thus, levels of phosphorylated CaMKII were examined for 3 sec and 300 sec inter-train intervals to assess whether PKA dependence was related to a decline in phosphorylated CaMKII with longer inter-train intervals. This peak was evaluated because experiments suggest that phosphoCaMKII anchors at the post-synaptic density (PSD) and is not accessible to dephosphorylation by protein phosphatase 1 [28]. This would imply that activity would be proportional to peak value, and the resulting slow decay of phosphoCaMKII precludes a reasonable calculation of the area under the curve.

Fig 2B shows that peak activity of phosphorylated CaMKII with 300 sec intervals is lower than with 3 sec intervals, which is opposite to the temporal sensitivity of PKA, suggesting that PKA activity is compensating for a frequency-dependent deficit in CaMKII. To further compare the CaMKII temporal sensitivity with the PKA temporal sensitivity, Fig 2C explores the phosphorylated activity of CaMKII for a range of inter-train intervals. PhosphoCaMKII decreases as temporal interval increases (beyond 3 sec), in agreement with experiments [29]. The time constant of this decrease is 20.8 sec, and phosphoCaMKII drops to 95% of its peak value with a 62 sec inter-train interval. The sum of (normalized) phosphoCaMKII and PKA activity is independent of interval for all but the very shortest intervals suggesting that PKA is required for spaced stimulation to compensate for a decrease in CaMKII. This result leads to the prediction that PKA will be required for inter-train intervals greater than ∼62 sec.

### Experimental test of critical temporal interval predicted by model

**Figure 3:**
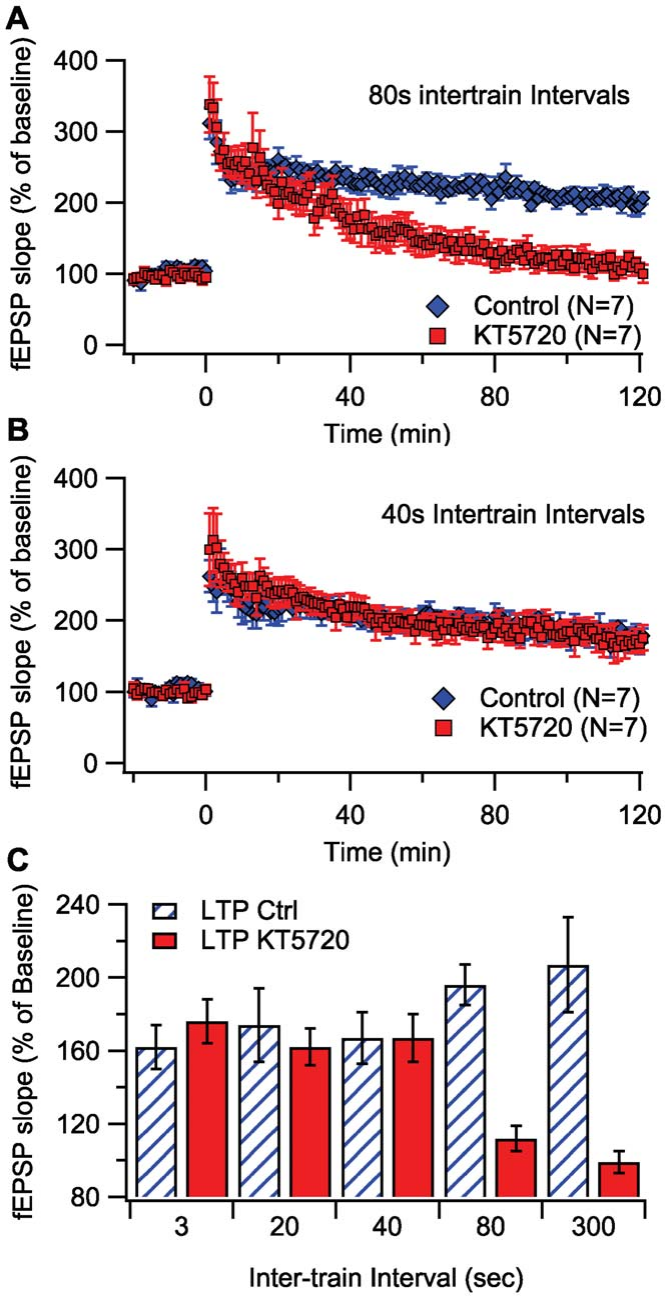
Experiments to verify that the critical inter-train interval for the PKA-dependence of L- LTP is ∼60 sec. (A) LTP induced using a 80 sec inter-train interval (n = 7) is blocked by KT5720 (*p*<0.05), which demonstrates PKA dependence. (B) In contrast, when using a 40 sec inter-train interval (bottom; n = 7), LTP in KT5720 slices is not different from vehicle control (*p*>0.05), demonstrating PKA independence. In both panels, fEPSPs for KT5720 treated slices are shown with red circles, and vehicle controls with blue circles. (C) Summary of temporal sensitivity of PKA dependence of L-LTP. Data for 3 sec, 20 sec and 300 sec are from [3].

The prediction that PKA is required for intervals greater than ∼62 sec was tested by inducing L-LTP at Schaffer collateral-CA1 synapses in mouse hippocampal slices using 4 trains of high frequency stimulation, with either 40 sec or 80 sec inter-train intervals, in the presence of either KT5720 or vehicle as control. As shown in Fig 3A, LTP induced by stimulation trains delivered at 80 sec inter-train intervals was attenuated in KT5720-treated slices compared to vehicle controls. At 120 min after LTP induction, the average fEPSP slopes were significantly different: 196±11% for vehicle-treated slices and 112±7% for KT5720-treated slices (Mann-Whitney *U* test, *p*<0.05). This demonstrates that LTP induced by 4 trains of high frequency stimulation delivered at 80 sec inter-train intervals requires PKA. In contrast, fEPSP slopes are not significantly different between KT5720 and control slices using 40 sec inter-train intervals (Fig 3B). At 120 min after LTP induction, the average fEPSP slopes were 167±14% for vehicle-treated slices and 167±13% for KT5720-treated slices (Mann-Whitney *U* test, *p*>0.05). This indicates that LTP stimulated by 4 trains of high frequency stimulation delivered at 40 sec inter-train interval is PKA-independent. These results, and previous experimental results on PKA dependence [3], are summarized in Fig 3C, which demonstrates that L-LTP induced with temporal intervals of 3 sec to 40 sec are PKA-independent, whereas L-LTP induced by temporal intervals of 80 sec and 300 sec are PKA-dependent. These experiments support the model prediction, thus verifying the model and its explanation of mechanisms underlying PKA dependence.

### The role of dopamine in L-LTP

**Figure 4:**
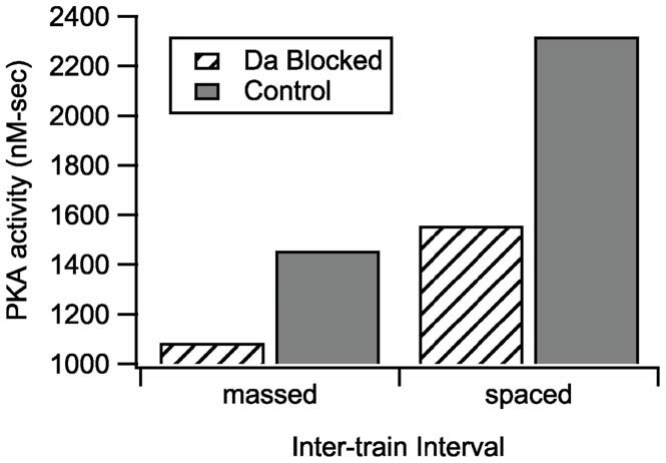
Simulations were repeated with the dopamine receptor blocked, to evaluate the contribution of dopamine to L-LTP. Cumulative PKA activity is reduced significantly with both massed and spaced stimulation intervals. The PKA activity with spaced stimulation and dopamine blocked is comparable to PKA activity with massed stimulation and dopamine unblocked. The reduction in PKA activity for the massed stimulation case is not functionally significant because PKA is not needed for L-LTP produced by massed stimulation.

In the hippocampus, adenylyl cyclase type 1 is synergistically activated by both calcium-calmodulin and dopamine, which is released during 100 Hz stimulation [30] from fibers innervating hippocampal area CA1 [31]. Further support for the role of dopamine is provided by experiments that show that L-LTP induced using a 10–12 min inter-train interval is reduced when dopamine receptors are blocked [8],[30],[32]. Thus, simulations were repeated with the dopamine receptor blocked, to evaluate the contribution of dopamine to L-LTP. Fig 4 shows that cumulative PKA activity is reduced significantly with both massed and spaced stimulation intervals when dopamine receptor function is blocked. The PKA activity for a 300 sec inter-train interval with no dopamine is similar to the PKA activity for the 3 sec inter-train interval with dopamine present, suggesting that L-LTP induction with spaced stimuli requires the higher PKA produced by spaced stimuli. Though the lack of dopamine reduces PKA activity for the 3 sec inter-train interval, this is not functionally significant because L-LTP with massed stimulation is PKA-independent. In other words, a 300 sec inter-train interval activates insufficient quantities of CaMKII, and additional dopamine stimulated PKA activity is required for the 300 sec interval only. Stimulation with a 3 sec interval activates sufficient CaMKII, and thus, the model predicts that blocking dopamine receptors would not block L-LTP for this interval.

### Mechanism underlying PKA temporal sensitivity

**Figure 5:**
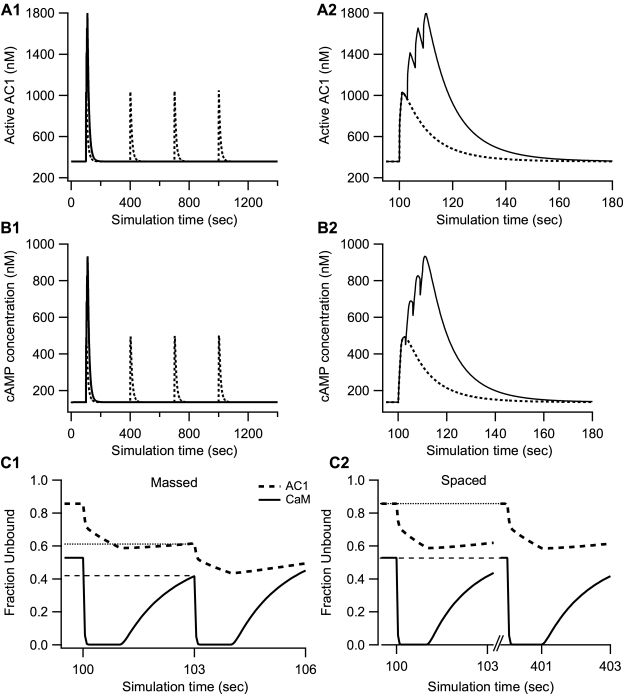
Mechanism underlying sensitivity of PKA activation to different temporal patterns of stimulation. In both (A) and (B) the left column (A1, B1) shows 1400 sec of simulation time and the right column (A2, B2) shows the first 180 sec to better show the four tetani of massed stimulation. (A) With massed trains, activation of adenylyl cyclase begins to saturate with the first train, leading to sublinear summation of adenylyl cyclase activity in response to subsequent trains; (B) cAMP concentration exhibits sub-linear summation with massed stimuli; thus less cAMP is produced with massed than with spaced trains. (C) Depletion of adenylyl cyclase and calmodulin contribute to the non-linear summation. Fractions of unbound adenylyl cyclase 1 and calmodulin are much lower for the second stimulus train for massed stimulation (C1), but not for spaced stimulation (C2). Dashed horizontal line allows comparison of unbound calmodulin between first and second train. Dotted horizontal line allows comparison of unbound adenylyl cyclase 1 between first and second train.

The sensitivity of cumulative PKA activity to different temporal intervals follows that of adenylyl cyclase (Fig 5A) and cAMP (Fig 5B). The first 100 Hz train produces a 600 nM increase in adenylyl cyclase activity from binding to calmodulin and G_sα_ (Fig 5A2)_._ With the massed protocol, the second 100 Hz train only produces an additional 300 nM increase in adenylyl cyclase activity, because free adenylyl cyclase is depleted with massed trains to a significant degree. More than 80% of unbound adenylyl cyclase 1 is available for activation by the first train of stimulation (Fig 5C); unbound adenylyl cyclase 1 decreases by 20% for massed (Fig 5C1), but remains at more than 80% for spaced stimulation (Fig 5C2). Calmodulin, which activates adenylyl cyclase 1, also exhibits a small degree of depletion, in part because it binds to other molecules, such as protein phosphatase 2B and phosphodiesterase 1B, with extremely high affinity. Thus, subsequent stimulation trains produce smaller increments in activated adenylyl cyclase for massed, but not for spaced stimulation. These lower adenylyl cyclase activity increments result in lower cAMP increments with subsequent trains using massed stimulation: 300 nM for the first train and 150 nM for the second train (Fig 5B2); thus the total cAMP produced from four trains of stimulation is less than four times the cAMP produced for one train. Note that the temporal pattern of cAMP, which decays within 40 sec to basal levels, agrees with measurements using a fluorescent Epac-1 probe [33],[34], verifying this aspect of the model. Therefore, the activation of PKA is greater with spaced as compared to massed stimulation because adenylyl cyclase activity is greater with spaced as compared to massed stimulation.

### Temporal sensitivity propagates to PKA targets

**Figure 6:**
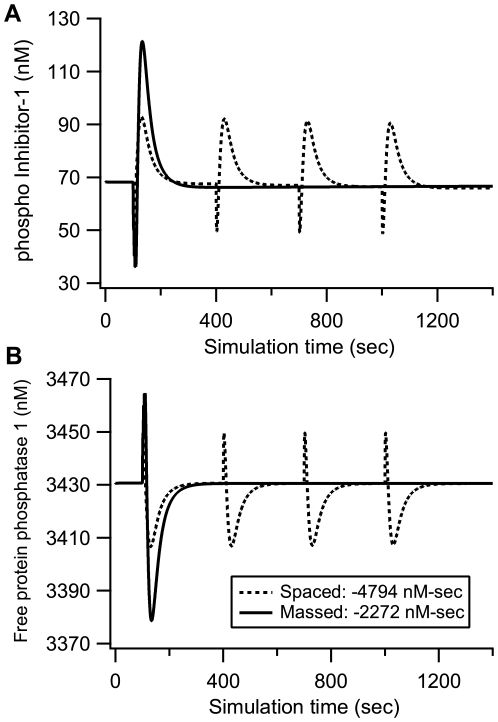
The increase in PKA activity due to spaced stimulation propagates to downstream targets inhibitor-1 and protein phosphatase 1. (A) The amount of phosphorylated inhibitor-1 is ∼50% higher for spaced than massed stimulation. The initial decrease in phosphorylated inhibitor-1 (below baseline) is due to activation of protein phosphatase 2B. (B) The sharp peaks of protein phosphatase 1 during stimulation are caused by the initial decrease of phosphorylated inhibitor-1. These transient increases are followed by longer periods of decrease in free protein phosphatase 1 below basal level, caused by an increase of phosphorylated inhibitor-1. This simulation shows the concentration of protein phosphatase 1 is reduced ∼50% more by the spaced stimulation protocol than the massed protocol (in the legend a negative value of area under the curve denotes a decrease in activity).

PKA is important in LTP because it phosphorylates AMPA receptors and inhibitor-1, as well as other plasticity related proteins [35]–[38], not all of which have been identified. Because rates of AMPA receptor phosphorylation have not been directly measured, we chose to evaluate the effect of PKA activity on a different target, namely inhibitor-1. Furthermore, inhibition of protein phosphatase 1 by phosphorylated inhibitor-1 will enhance phosphorylation of many PKA targets via inhibition of dephosphorylation. Thus, examination of the phosphorylation state of inhibitor-1 in these simulations both represents the ability of PKA to phosphorylate downstream targets, and also indicates whether free protein phosphatase 1 will be sensitive to temporal interval. As seen in Fig 6A, the amount of phosphorylated inhibitor-1 is 50% greater for spaced than massed stimulation. Similar to that observed with PKA activity, the peak value is higher for massed stimuli, but total phosphorylated inhibitor-1 is greater for spaced stimuli. This shows that the temporal sensitivity of PKA activity propagates to downstream targets. The phosphorylated inhibitor-1 binds to protein phosphatase 1 with high affinity, inhibiting its activity. Thus, the 50% increase in phosphorylated inhibitor-1 produces a 50% decrease in protein phosphatase 1 (Fig 6B). This suggests that the enhanced activity of PKA with spaced stimulation will suppress protein phosphatase 1 activity, reinforcing the phosphorylation of plasticity related proteins. To test whether the enhanced inhibitor-1 phosphorylation increased CaMKII phosphorylation, simulations were repeated with PKA phosphorylation of inhibitor-1 blocked. The decrease in CaMKII phosphorylation was small (Fig S2A), suggesting other mechanisms to enhance PKA activity are important (discussed below).

### Robustness of the model

To investigate the robustness of results (i.e., whether the results are sensitive to variation in parameters), simulations are repeated using parameter values 2 to 10 times larger or smaller than the control values, for parameters that are least constrained by biochemical data. For instance, though the quantity of PKA has been estimated to be 1.2 µM in brain tissue, assuming the protein distributes in 70% of intercellular space [39], the existence of localized pools of PKA suggest that the effective quantity of this enzyme in the synapse could be higher than the estimated quantity. Similar arguments can be made for protein phosphatase 1. Thus, simulations are repeated using both higher and lower quantities of PKA, protein phosphatase 1, protein phosphatase 2B, as well as Ca^2+^ influx.

As shown in Fig S3, the main results from this model are qualitatively robust. Though the PKA activity increases when enzyme quantities are increased, spaced stimulation still produces ∼60% more total activity than massed stimulation (Fig S3A). The quantity of protein phosphatase 1 has no effect on PKA activity, but does modify the decay rate of phosphoCaMKII. Regardless of protein phosphatase 1 quantity or dephosphorylation rate, spaced stimulation produces lower phosphorylated CaMKII than massed stimulation (Fig S3B). Peak Ca^2+^ has a different effect on phosphoCaMKII: it changes the peak value with no change in decay, and no change in frequency sensitivity (Fig S3D). PKA was minimally affected by variation of peak Ca^2+^ (Fig S3C).

A recent FRET imaging experiment suggests that CaMKII activity in spines is transient in response to synaptic stimulation [40]. Thus, additional simulations evaluated whether the results are sensitive to persistence of CaMKII. Transient phosphoCaMKII was produced by allowing protein phosphatase 1 to dephosphorylate the calmodulin bound form of phosphoCaMKII (Fig S2A). Using this more transient phosphoCaMKII in simulations, CaMKII activity is quantified as area under the curve (instead of peak). Fig S2B shows that area under the curve increases for PKA and decreases for phosphoCaMKII with increasing inter-train interval, the latter with a time constant of 17.8 sec – close to the time constant for the persistent model of CaMKII. Thus, the prediction that PKA is required to compensate for a decrease in phosphoCaMKII is robust to this variation in CaMKII dynamics.

## Discussion

### Temporal spacing of synaptic stimulation alters the PKA-dependence of L-LTP

To better understand the complex intracellular signaling networks underlying the temporal sensitivity of PKA dependence of L-LTP, we developed a computational model of the calcium and cAMP signaling pathways involved in PKA and CaMKII activation in hippocampal CA1 neurons. The model is based on published biochemical measurements of many key signaling molecules, most notably PKA and CaMKII. Simulations of four trains of 100 Hz stimuli separated by 300 sec or 3 sec revealed that spaced stimulation activates more PKA and less CaMKII than massed stimulation. Thus, PKA activity may be required for spaced stimulation because more of it is active, and less phosphoCaMKII is available. Simulations were repeated for a range of inter-train intervals, to further explore the PKA dependence of L-LTP induction. PKA activity increases exponentially with increasing inter-train interval, compensating for the decrease in phosphoCaMKII with increasing inter-train interval. The time constant of phosphoCaMKII decrease was 20.8 sec; thus, the model predicts that L-LTP induced with an inter-train interval greater than 62 sec (3τ) will be dependent on PKA, and L-LTP induced with an interval less than 62 sec will be independent of PKA. Experiments confirm this prediction, showing that a 40 sec inter-train interval is PKA-independent and an 80 sec inter-train interval requires PKA.

The temporal sensitivity of PKA differs from that in a previously published model [29] mainly due to the different method of quantifying PKA activity. The present study measured cumulative PKA activity as area under the curve and found an increase with temporal interval. In the previously published model, PKA activity was quantified as the peak activity at 600 sec after the last tetanus, to compare with experimental measurements which also measured activity at 600 sec after the last tetanus. In that study, PKA peak activity did not exhibit temporal sensitivity, and thus could not explain the temporal sensitivity of PKA dependence of LTP. To compare the present model results with that previous model, PKA activity was quantified as activity at 600 sec after the last tetanus. Using this quantification, temporal sensitivity of PKA activation in the present model is minimal, in agreement with Ajay and Bhalla [29]. Nonetheless, cumulative activity is a better measure of the ability of a kinase to phosphorylate downstream substrates such as AMPA receptors or inhibitor-1, because cumulative activity is proportional to average enzyme activity over the time course of the enzyme. With regards to CaMKII activity, both cumulative, when CaMKII phosphorylation is transient, and peak when CaMKII phosphorylation is persistent, were good predictors of the critical inter-train interval.

Another PKA-dependent form of L-LTP is induced by theta-burst stimuli [41], which uses short bursts of 100 Hz stimulation (e.g., 4 pulses) repeated at 200 msec intervals. A typical experimental induction protocol uses fifteen repetitions of 4 bursts yielding 60 pulses total, far less than provided with 4 bursts of 100 Hz. Model simulations show that both CaMKII and PKA are lower with this stimulation due to the lower number of pulses. These simulations of post-synaptic mechanisms cannot explain the PKA-dependence of theta-burst L-LTP because theta-burst L-LTP involves pre-synaptic mechanisms [42].

### Mechanisms increasing localized PKA activity

In our model, activated PKA is represented as the cumulative quantity of the free catalytic subunit. Although stimulation produces about a 60% increase in free catalytic subunit, the peak quantity of free catalytic subunit is relatively small (less than 50 nM for massed stimulation and less than 35 nM for spaced stimulation). This may suggest that the quantity of free catalytic subunit would be insufficient for the PKA-dependent L-LTP (i.e., both the increase in inhibitor-1 phosphorylation, and the inhibition in CaMKII dephosphorylation were small), especially given the number of PKA targets. The small quantity of PKA free catalytic subunit produced is due to the high affinity (9 nM) of the regulatory subunit for the catalytic subunit even when all four cAMP molecules are bound. One possible solution to the low quantity of PKA catalytic subunit is that the cAMP-saturated holoenzyme is catalytically active toward its substrates. Binding of four cAMP to the linker region of the regulatory subunit causes a conformational change, exposing the catalytic site without complete dissociation [43],[44]. The L-LTP induction paradigms produce a significant amount of cAMP-saturated holoenzyme (twice as much as free catalytic subunit). If this form is active, the quantity of active PKA would be three times higher. In addition, the actions of anchoring also increase local PKA activity in the synapse. A kinase anchoring proteins (AKAPs) bind to the regulatory subunit of the PKA holoenzyme [45]. By tethering the PKA holoenzyme near a preferred substrate at a particular subcellular location, a small number of molecules could produce significant phosphorylation of its substrate. In support of this concept, experiment shows that hippocampal synaptic plasticity requires not only PKA activation, but also the activation of an appropriately anchored pool of PKA [42],[46].

### Competition for Calmodulin limits PKA and CaMKII activation

As previously mentioned, the conceptual model of CaMKII activation predicts a positive feedback loop in which increased phosphorylation leads to an increased rate of subsequent phosphorylation. Therefore, subsequent stimulus trains should produce increasing increments in CaMKII activity. Similar to other single compartment models [19],[20],[47], this positive feedback response is not observed in the model unless additional calmodulin is provided (Fig S4A). Calmodulin binds with high affinity to protein phosphatase 2B and phosphodiesterase 1B, and with intermediate affinity to adenylyl cyclase as well as CaMKII. This binding causes a decrease in Ca_4_-calmodulin with subsequent trains due to competition for calmodulin between the CaMKII pathway and other pathways. Calmodulin is a diffusible protein; thus, in a dendritic spine free calmodulin would diffuse into the spine from the dendrite to replace the bound calmodulin. In addition, neurogranin is a calmodulin binding protein that releases calmodulin upon Ca^2+^ stimulation; in essence neurogranin acts as a calmodulin reservoir [48]–[50]. Simulations in which additional calmodulin is provided yields a frequency sensitivity of phosphoCaMKII that agrees with experimental measurements [29].

Not only CaMKII, but also PKA activation is limited by free available calmodulin, since the predominant adenylyl cyclases (1/8) in hippocampus are activated by calmodulin. Calmodulin depletion results in decreasing increments of adenylyl cyclase activity, cAMP production, and PKA activation with massed stimulation, causing sublinear summation. Providing additional calmodulin reduces the degree of sublinear summation, though the limited quantity of adenylyl cyclase 1 and adenylyl cyclase 8 also contributes to sublinear summation. Thus, as illustrated in Fig S4B, the incorporation of additional calmodulin does not change the main result, namely that PKA cumulative activity is higher with spaced stimulation.

### PKA phosphorylation substrates involved in LTP

One way in which LTP is expressed post-synaptically is as enhanced phosphorylation of AMPA receptors leading to insertion of new AMPA receptors. The phosphorylation state of AMPA receptors depends on the balance of kinases and phosphatases including PKA, CaMKII and protein phosphatase 1 [51]–[53]. Active PKA directly phosphorylates the AMPA receptor GluR1 subunit at Ser845, enhancing AMPA channel function [54] and leading to increased AMPA channel expression. PKA indirectly governs the dephosphorylation activity of protein phosphatase 1 by phosphorylating inhibitor-1 with very high affinity allowing it to bind protein phosphatase 1. Other substrates of PKA are implicated in hippocampal synaptic plasticity, including phosphodiesterase type 4D3 and inositol triphosphate receptor channels [55],[56].

AMPA channel phosphorylation modulates expression of LTP, but transcription and translation are required for L-LTP [57]. A target of phosphorylation by active PKA involved in transcription is the cAMP Response Element Binding Protein (CREB) in the nucleus. Phosphorylated CREB increases activation of transcription and protein translation. Members of the mitogen activated protein kinase (MAPK) family are targets of PKA that plays a role in transcription, translation, and synaptic plasticity [58]. One member of the MAPK family is extracellular signal-regulated kinase type II, which is phosphorylated by several signaling pathway kinases, such as PKA and also CaMKII through synGAP [59],[60]. Ajay and Bhalla [29] demonstrate that both extracellular signal-regulated kinase type II activity and the magnitude of LTP induction are maximal using inter-train intervals of 300–600 sec; in this context, our results suggest that part of the temporal dependence of extracellular signal-regulated kinase type II is due to PKA. Yet another target of PKA involved in maintenance of LTP is the atypical protein kinase C, type Mζ, which is phosphorylated at a site of convergence of both PKA and CaMKII [61]. Thus, our hypothesis that the combination of CaMKII plus PKA is critical for L-LTP is consistent with several of these target proteins whose activity integrates multiple kinases.

Additional evidence suggests that PKA is critical for synaptic tagging [46],[62],[63], which provides the synaptic specificity important for information processing. The synaptic tag theory proposes that L-LTP associated gene products can only be captured and utilized at synapses that have been tagged by previous activity [64]. Both CaMKII and PKA have been implicated in phosphorylation of an unidentified synaptic substrate, which appears necessary to set a tag at activated synapses to allow capture of plasticity factors (i.e. CRE-driven gene products, newly synthesized AMPA receptors or mRNAs). One possibility is that phosphorylation of the tag can be provided by either CaMKII, PKA, or both, depending on the temporal interval of stimulation.

### Spatial aspects of signaling and LTP

To further evaluate L-LTP, it will be necessary to include some of these signaling events downstream of PKA, such as activation of extracellular signal-regulated kinase type II. Furthermore, anchoring of proteins in spines, communication with the larger dendrites, and other spatial details all suggest that single compartment models are not sufficiently accurate. Thus, multi-compartmental models will be critical for evaluating issues such as the distribution of synaptic inputs underlying the spread of biochemical signals from synapses to dendrites [25] or the diffusion of biochemical signals between spines [65]. For example, preliminary simulations using a multi-compartmental stochastic model suggest that localization of dopamine receptors and PKA leads to larger phosphorylation of inhibitor-1, and inhibition of protein phosphatase 1, as experimentally observed [35]. Given the complexity of non-linear interactions among signaling pathways, simulations using these novel multi-compartmental models promise to enhance understanding of the mechanisms underlying synaptic plasticity.

## Methods

### Ethics statement

All research with animals was consistent with NIH guidelines and approved by the IACUC at the University of Pennsylvania.

### Computational model

The single compartment, computational model, illustrated in Fig 1A, consists of signaling pathways known to underlie synaptic plasticity in hippocampal CA1 pyramidal neurons. Calcium influx through the NMDA receptor leads to calcium-calmodulin activation of adenylyl cyclase types 1 and 8 [66], phosphodiesterase type 1B, protein phosphatase 2B (PP2B or calcineurin) and CaMKII. In addition, CA1 is innervated by dopamine fibers[67], and dopamine type D1/D5 receptors, coupled to G_sα_, are expressed in CA1[68]. Dopamine levels increase in response to 100 Hz stimulation [30], leading to enhanced adenylyl cyclase (type 1) activity [69],[70], and increases in cAMP, which activate PKA [71],[72]. The phosphorylation of inhibitor-1 by PKA transforms inhibitor-1 into a potent inhibitor of protein phosphatase 1 [73],[74], thereby decreasing CaMKII dephosphorylation. Though not included in the model, the phosphorylation state of the AMPA receptor is controlled by CaMKII, PKA and protein phosphatase 1 [75],[76].

All reactions in the model are listed in Tables 1 and 2 and are described as bimolecular chemical reactions or as enzymatic reactions except for PKA (described below) and CaMKII reactions (Text S1). A set of rate equations is constructed to describe the biochemical reactions of the model's pathways. These rate equations are nonlinear ordinary differential equations with concentrations of chemical species as variables. Equations are derived assuming all reactions are in a single compartment and the number of molecules is sufficient for mass action kinetics, as follows:

For a bimolecular chemical reaction:
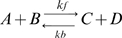
(1)


in which substrates, A and B, are consumed to create products, C and D, the rate of reaction is represented by a differential equation of the form
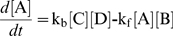
(2)


where *k_f_* and *k_b_* are the forward and backward rate constants of the reaction, and *K_d_* = *k_f_* /*k_b_* is the affinity.

For an enzyme-catalyzed reaction:
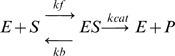
(3)


where E, S, ES and P denote enzyme, substrate, enzyme-substrate complex and product, the rate of production of P, *d*[P]/*dt,* is given by: 
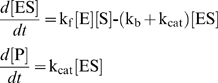
(4)


For enzymatic reactions *k_ca_*_t_ defining the last, catalytic step, is the rate at which product appears, and the affinity *K*_m_ is defined as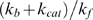. When *k_b_* is not known explicitly, *k_b_* is defined as 4 times *k_cat_* [14].

PKA (cAMP-dependent protein kinase) is activated by the cooperative binding [77] of cAMP to two tandem cAMP-binding sites (called A and B sites) on each of the two regulatory subunits. The binding of four cAMP leads to the dissociation of the active catalytic subunits, allowing them to phosphorylate their protein targets [4]. In the model pairs of cAMP bind with first order kinetics as measured by the fraction of free catalytic subunit as a function of cAMP concentration [78],[79]. The affinity of site A relative to the affinity of site B is obtained from Herberg et al. [77],[80]. Keeping this ratio, the affinity of these sites was adjusted to match the overall affinity of the holoenzyme [72] (Fig S1A).

The only exception to the single compartment approximation is that additional calmodulin was provided to prevent calmodulin from decreasing significantly during stimulation. Calmodulin is a diffusible protein, thus in a dendritic spine, free calmodulin would diffuse into the spine from the dendrite to replace the bound calmodulin. In addition, neurogranin is a calmodulin binding protein that releases calmodulin upon Ca^2+^ stimulation; in essence neurogranin acts as a calmodulin reservoir [48]–[50]. The increase in calmodulin was made proportional to the difference between initial calmodulin and free calmodulin. The rationale is that without additional calmodulin, subsequent stimulation trains produce a smaller increment in phosphoCaMKII (1^st^: 166 nM, 4^th^: 154 nM), which is inconsistent with the positive feedback loop implicit in a widely accepted conceptual model of CaMKII activation [81],[82]. With added calmodulin, increased phosphorylation leads to an increased rate of subsequent phosphorylation since phosphorylated CaMKII has higher activity than calmodulin-bound unphosphorylated CaMKII (Fig S4A).

Rate constants used in this model were obtained from the biochemical literature and are tabulated with their reactions in Tables 1 and 2. The differential equations are programmed in XPPAUT and run under the Linux operating system. The model is freely available for download from ModelDB: http://senselab.med.yale.edu/senselab/modeldb/ Simulations used the numerical integration method called “stiff” with a time step of 0.01 sec.

### Stimulation protocols

To understand how L-LTP dependence on PKA is sensitive to temporal interval, simulations were performed using stimulation paradigms identical to the experimental paradigms typically used to induce L-LTP. Thus, we simulated four trains of 100 Hz stimulation for a duration of 1 sec (Fig 1B), delivered either 3 sec apart (massed) or 300 sec apart (spaced), and compared these two simulation “groups” with a control simulation run without induction stimuli. The total number stimulation pulses may modify the activation of PKA or CaMKII; therefore, they were held constant for these simulations [3],[83]. Each stimulation pulse triggered a transient elevation in intracellular calcium concentration (Fig 1B), similar to experimental observations [84],[85] of the response to synaptic activation of the NMDA receptors. Stimulation at 100 Hz frequency results in accumulation of calcium (Fig 1C) [86] because individual transients do not completely decay. Each train of stimulation was accompanied by an increase in dopamine concentration, as if released from dopaminergic synaptic terminals. The amplitude of the dopamine pulse (Fig 1C) is based on Rice and Cragg [87]. The simulation was run for a period of 3000 seconds following stimulation.

### Experimental methods

Hippocampal slices were prepared from wild type mice as described previously [9]. Extracellular field excitatory postsynaptic potentials (fEPSPs) were recorded with a glass microelectrode positioned in stratum radiatum of area CA1. Evoked fEPSPs were elicited by stimulation of the Schaeffer collateral fibers with an extracellular bipolar nickel-chromium electrode. The stimulation intensity was adjusted to give fEPSP amplitudes that were approximately 40% of maximal fEPSP sizes. Control “baseline” responses were elicited once per minute at this intensity. The stimulation protocol was applied by delivering four trains (100 Hz, each of 1sec duration) with either 40 sec or 80 sec inter-train intervals in either pretreated KT5720 hippocampal slices or control (Vehicle) slices. KT5720 (Biomol), an inhibitor of catalytic subunits of PKA [88], was dissolved in DMSO and diluted in ACSF to a final perfusate concentration of 1 µM (0.1% DMSO), the control slices were pretreated with 0.1% DMSO vehicle, which had no effect on basal fEPSPs. KT5720 or vehicle was delivered for 30 minutes, from 15 minutes before, until 15 minutes after LTP induction.

Data analysis was performed using Statistica 7.1 software (Statsoft, Inc., Tulsa, OK). Electrophysiological data from LTP recordings were analyzed by nonparametric tests because the analyses of repetitive recordings over long durations do not allow the use of parametric tests [89]. The initial slope of the fEPSP at each time point was analyzed. The Mann-Whitney *U* test was used to compare between two groups. Differences were considered statistically significant when *p*<0.05.

## Supporting Information

Text S1 Methods and Model Robustness. Exposition of model reactions, and source of parameters; demonstration that model results are robust to parameter variations
(0.09 MB DOC)

Figure S1 Models of PKA and CaMKII activation reproduce experimental observations (A) Schematic representation of PKA activation. R: regulatory subunit, C: catalytic subunit. (B) Schematic representation of CaMKII activation by Ca4-calmodulin (4Ca2+-CaM) and autophosphorylation. CK: unbound CaMKII, CKCam: CaMKII bound with Ca4-calmodulin, CKpCam: CaMKII autophosphorylated at T286 with Ca4-calmodulin trapped, and CKp: autonomous CaMKII in which Ca4-calmodulin dissociates but the subunit remains phosphorylated at T286. (C) Percent activation of PKA holoenzyme versus cAMP concentration for model agrees with experiments. (D) The frequency sensitivity of CaMKII compares with the experimental results from De Koninck and Schulman [16]. For each frequency, 100 pulses are given. The pulse duration is 200 ms, with 500 µM of Ca2+ and 100 nM calmodulin.
(1.56 MB EPS)

Figure S2 Simulations showing that results are insensitive to persistence of CaMKII phosphorylation. (A) Transient phosphoCaMKII, suggested by recent FRET imaging [20], was produced by allowing protein phosphatase 1 to dephosphorylate the calmodulin bound form of phosphoCaMKII. The decrease in CaMKII phosphorylation is very small when PKA activity is blocked, suggesting the importance of spatial mechanisms or saturated holoenzyme activity for enhancing the effect of PKA. (B) Using this more transient phosphoCaMKII, CaMKII activity is quantified as area under the curve (instead of peak). Cumulative activity of phosphoCaMKII decreases with increasing inter-train interval and cumulative activity for PKA increases with increasing inter-train interval.
(0.62 MB EPS)

Figure S3 Results are robust to variation in parameters. (A) Variation of PKA enzyme quantity: both basal and peak PKA activity varied but spaced stimulation produces ∼60% more total activity than massed. (B) Effect of protein phosphatase 1 enzyme quantities on CaMKII phosphorylation. The peak values of active CaMKII are increased slightly with lower protein phosphatase 1 quantity, because less protein phosphatase 1 dephosphorylation occurs. None the less, in all cases spaced stimulation produces larger CaMKII phosphorylation than massed stimulation. (C) CaMKII activity is greater with higher calcium concentration and smaller with lower calcium concentration. In all cases, CaMKII activity with spaced stimulation is less than CaMKII activity with massed stimulation. (D) Cumulative PKA activity depends on calcium concentration, but in all cases PKA activity with spaced stimulation is greater than PKA activity with massed stimulation.
(0.32 MB EPS)

Figure S4 Effect of additional calmodulin on PKA and CaMKII activity. (A) Without additional calmodulin, subsequent stimulation trains produce a smaller increment in phosphoCaMKII. With added calmodulin, increased phosphorylation leads to an increased rate of subsequent phosphorylation, because phosphorylated CaMKII has higher activity than unphosphorylated CaMKII (Fig S4A). This response of CaMKII with added calmodulin (increasing increments with subsequent stimulation trains) is consistent with the positive feedback loop implicit in a widely accepted conceptual model of CaMKII activation. (B) The incorporation of additional calmodulin does not change the main result, namely that PKA cumulative activity is higher with spaced stimulation.
(0.27 MB EPS)
