# Supplementary material for: Temporal Sensitivity of Protein Kinase A Activation in Late-Phase Long Term Potentiation
Source: PLoS Comput Biol. 2010 Feb 26;6(2):e1000691. doi: 10.1371/journal.pcbi.1000691 (PMC2829045; doi:10.1371/journal.pcbi.1000691)
Supplement: Text S1 — Methods and Model Robustness. Exposition of model reactions, and source of parameters; demonstration that model results are robust to parameter variations (0.09 MB DOC) [file pcbi.1000691.s001.doc]

**Temporal sensitivity of protein kinase A activation in late phase long term potentiation**

MyungSook Kim, Ted Huang, Ted Abel and Kim T. Blackwell

**Supplemental Methods:**

***Description of reactions included in model and source of parameters***

Reactions and rate constants in the model were obtained as follows:

**Glutamate and calcium activated enzymes**

Glutamate is modeled by its effect on calcium, thus glutamate is simulated as an increase in intracellular calcium concentration. Calcium binds to calmodulin, which has four calcium binding sites: two N sites (fast, high affinity) and two C sites (slow, low affinity) [1]. Calcium binds to calmodulin in pairs, producing the active Ca_4_-calmodulin in two consecutive steps. In addition to CaMKII, Ca_4_-calmodulin molecule binds to and activates several enzymes in hippocampal CA1 neurons. Protein phosphatase 2B has a very high affinity (28 pM) for Ca_4_-calmodulin [2]. Activated protein phosphatase 2B dephosphorylates inhibitor-1 that is phosphorylated by active PKA [3] on Thr 35. Other enzymes activated by Ca_4_-calmodulin are described below.

**Neurotransmitters (Dopamine/ Norepinephrine) and G-protein coupled receptor**

In a hippocampal CA1 neuron, the postsynaptic dopamine D1 [4] and β-adrenergic receptors are coupled to the G_sα_ type of GTP-binding protein. This stimulatory G protein (G_sα_GTP) activates adenylyl cyclase. The Ca^2+^-stimulated adenylyl cyclases, types 1 and 8 are abundantly expressed in hippocampus. Adenylyl cyclase 1 is synergistically stimulated by Ca^2+^ and β-adrenergic/dopaminergic agonists and functions as a coincidence factor to integrate Ca^2+^ and receptor-mediated signals [5]. Adenylyl cyclase 8 is stimulated by Ca_4_-calmodulin but its Ca^2+^ sensitivity is approximately 5-fold lower than adenylyl cyclase 1 (half-maximal activation by Ca^2+^ is 150 nM for adenylyl cyclase 1 and 800 nM for adenylyl cyclase 8) [6].

**Cyclic AMP (cAMP) formation and PKA activation**

Activated adenylyl cyclases types 1 and 8 produce cyclic adenosine monophosphate (cAMP) from adenosine triphosphate (ATP). ATP is only modeled explicitly in the formation of cAMP and not in any other enzymatic phosphorylation step. cAMP is degraded into AMP by constitutively active phosphodiesterase type 4 [7] and phosphodiesterase type 1B in the hippocampus. Phosphodiesterase type 1B is activated by Ca_4_-calmodulin with an affinity of 0.28 nM [8]. cAMP binds to and activates cAMP-dependent protein kinase (PKA), which is a heterotetramer comprised of two regulatory subunits and two catalytic subunits. Each regulatory subunit has two binding sites for cAMP, one with high affinity and one with low affinity [9]. RIIβ is the major regulatory subunit isoform and is present CA1 [10]. The binding of two cAMP molecules to each regulatory subunit of PKA leads to the release of the catalytic subunits, representing the active PKA molecules. Parameters for cAMP binding and release of the catalytic subunits are adjusted in the model to reproduce the activation curve for the holoenzyme PKA [11], and the activation rate observed using Fluorescence Resonance Energy Transfer (FRET) imaging [12]. Active PKA phosphorylates inhibitor-1, which then inhibits protein phosphatase 1 with an affinity of 1 to 3 nM [13,14].

***CaMKII model***

The equations describing Ca_4_-calmodulin activation of CaMKII are a slight modification of a previously published model [15] that agrees quantitatively with experimental data [16]. The reactions in figure S1C model the transition of CaMKII subunits between 4 different states: unbound (CK); bound with Ca_4_-calmodulin (CKCam); autophosphorylated at T^286^ with Ca_4_-calmodulin trapped (CK_p_Cam); and autonomous (CK_p_) in which Ca_4_-calmodulin dissociates but the subunit remains phosphorylated at T^286^. Note that this model has a single trapped state, and Ca_4_-calmodulin dissociates as a unit from phoshorylated CaMKII. In contrast, Dupont’s CaMKII model has two trapped forms: Ca_4_-calmodulin bound to CaMKII releases Ca^2+^ first, after which apo calmodulin dissociates. Note that these two alternatives cannot be distinguished experimentally [17], and simulated CaMKII activity does not change if Ca_4_-calmodulin dissociation is modeled as two steps. The autophosphorylation rate V_a_ is similar to Dupont’s but eliminates the contribution from the autonomously active form (CK_p_) both for simplicity and because its contribution is minimal:

$$Va=Ka\times\left((CKCam)^{2}+(CKCam)\times(CK_{p}Cam)\right)$$

where Ka=$Ka'\times(CKCaM)$ and $Ka'=0.46$. This third order dependence on bound CaMKII subunits represents the fact that subunits need to be adjacent within a holoenzyme to phosphorylate each other. This modified model reproduces the experimental results [16] demonstrating that in the range of 1-10 Hz, a higher frequency of calcium pulses produces a higher activation of CaMKII. Our model of CaMKII activation by Ca_4_-calmodulin reproduces these experimental results (Fig S1D). In hippocampal slices, phosphoCaMKII measured at 600 sec after L-LTP induction was lower for a 600 sec inter-train interval than for a 20 sec inter-train interval [18]. Our simulations also replicate this observation, further validating our model of CaMKII activation.

**Supplemental Results**

***Robustness of results to parameter variations***

Dephosphorylation of protein phosphatase 1 in the model is limited to the autonomous form of phosphoCaMKII, though experiments suggest the possibility that protein phosphatase 1 also dephosphorylates the trapped (Ca_4_-calmodulin bound) form of phosphoCaMKII. To evaluate robustness of the results to this aspect of the model, simulations were repeated with the additional dephosphorylation reaction of trapped phosphoCaMKII, and with rate constants that were lower but still within the range of biochemical measurements. Similar to other robustness simulations, PKA and CaMKII exhibit qualitatively the same temporal sensitivity: PKA activity increases with inter-train interval, and CaMKII activity decreases with inter-train interval. In all reported simulations, the non-stimulated autonomous activity was 5% of total CaMKII activity [19]; but similar results were observed with higher and lower initial autonomous activity.

**Supplemental Figures**:

Figure S1. Models of PKA and CaMKII activation reproduce experimental observations

(A) Schematic representation of PKA activation. R: regulatory subunit, C: catalytic subunit. (B) Schematic representation of CaMKII activation by Ca_4_-calmodulin (4Ca^2+^-CaM) and autophosphorylation. CK: unbound CaMKII; CKCam: CaMKII bound with Ca_4_-calmodulin; CK_p_Cam: CaMKII autophosphorylated at T^286^ with Ca_4_-calmodulin trapped; and CK_p_: autonomous CaMKII in which Ca_4_-calmodulin dissociates but the subunit remains phosphorylated at T^286^. (C) Percent activation of PKA holoenzyme versus cAMP concentration for model agrees with experiments. (D) The frequency sensitivity of CaMKII compares with the experimental results from De Koninck and Schulman [16]. For each frequency, 100 pulses are given. The pulse duration is 200 ms, with 500 µM of Ca^2+^ and 100 nM calmodulin.

Figure S2: Simulations showing that results are insensitive to persistence of CaMKII phosphorylation. (A) Transient phosphoCaMKII, suggested by recent FRET imaging [20], was produced by allowing protein phosphatase 1 to dephosphorylate the calmodulin bound form of phosphoCaMKII. The decrease in CaMKII phosphorylation is very small when PKA activity is blocked, suggesting the importance of spatial mechanisms or saturated holoenzyme activity for enhancing the effect of PKA. (B) Using this more transient phosphoCaMKII, CaMKII activity is quantified as area under the curve (instead of peak). Cumulative activity of phosphoCaMKII decreases with increasing inter-train interval and cumulative activity for PKA increases with increasing inter-train interval.

Figure S3. Results are robust to variation in parameters. (A) Variation of PKA enzyme quantity: both basal and peak PKA activity varied but spaced stimulation produces ~60% more total activity than massed. (B) Effect of protein phosphatase 1 enzyme quantities on CaMKII phosphorylation. The peak values of active CaMKII are increased slightly with lower protein phosphatase 1 quantity, because less protein phosphatase 1 dephosphorylation occurs. None the less, in all cases spaced stimulation produces smaller CaMKII phosphorylation than massed stimulation. (C) CaMKII activity is greater with higher calcium concentration and smaller with lower calcium concentration. In all cases, CaMKII activity with spaced stimulation is less than CaMKII activity with massed stimulation. (D) Cumulative PKA activity depends on calcium concentration, but in all cases PKA activity with spaced stimulation is greater than PKA activity with massed stimulation.

Figure S4. Effect of additional calmodulin on PKA and CaMKII activity. (A) Without additional calmodulin, subsequent stimulation trains produce a smaller increment in phosphoCaMKII. With added calmodulin, increased phosphorylation leads to an increased rate of subsequent phosphorylation, because phosphorylated CaMKII has higher activity than unphosphorylated CaMKII (Fig S4A). This response of CaMKII with added calmodulin (increasing increments with subsequent stimulation trains) is consistent with the positive feedback loop implicit in a widely accepted conceptual model of CaMKII activation. (B) The incorporation of additional calmodulin does not change the main result, namely that PKA cumulative activity is higher with spaced stimulation.

References

1. Putkey JA, Kleerekoper Q, Gaertner TR, Waxham MN (2003) A new role for IQ motif proteins in regulating calmodulin function. J Biol Chem 278: 49667-49670.

2. Quintana AR, Wang D, Forbes JE, Waxham MN (2005) Kinetics of calmodulin binding to calcineurin. Biochem Biophys Res Commun 334: 674-680.

3. Mulkey RM, Endo S, Shenolikar S, Malenka RC (1994) Involvement of a calcineurin/inhibitor-1 phosphatase cascade in hippocampal long-term depression. Nature 369: 486-488.

4. Granado N, Ortiz O, Suarez LM, Martin ED, Cena V, Solis JM, Moratalla R (2008) D1 but not D5 dopamine receptors are critical for LTP, spatial learning, and LTP-Induced arc and zif268 expression in the hippocampus. Cereb Cortex 18: 1-12.

5. Wang H, Storm DR (2003) Calmodulin-regulated adenylyl cyclases: cross-talk and plasticity in the central nervous system. Mol Pharmacol 63: 463-468.

6. Nielsen MD, Chan GC, Poser SW, Storm DR (1996) Differential regulation of type I and type VIII Ca2+-stimulated adenylyl cyclases by Gi-coupled receptors in vivo. J Biol Chem 271: 33308-33316.

7. Harada N, Nishiyama S, Ohba H, Sato K, Kakiuchi T, Tsukada H (2002) Age differences in phosphodiesterase type-IV and its functional response to dopamine D1 receptor modulation in the living brain: a PET study in conscious monkeys. Synapse 44: 139-145.

8. Herman SB, Juilfs DM, Fauman EB, Juneau P, Menetski JP (2000) Analysis of a mutation in phosphodiesterase type 4 that alters both inhibitor activity and nucleotide selectivity. Mol Pharmacol 57: 991-999.

9. Herberg FW, Taylor SS, Dostmann WR (1996) Active site mutations define the pathway for the cooperative activation of cAMP-dependent protein kinase. Biochemistry 35: 2934-2942.

10. Licameli V, Mattiace LA, Erlichman J, Davies P, Dickson D, Shafit-Zagardo B (1992) Regional localization of the regulatory subunit (RII beta) of the type II cAMP-dependent protein kinase in human brain. Brain Res 578: 61-68.

11. Zawadzki KM, Taylor SS (2004) cAMP-dependent protein kinase regulatory subunit type IIbeta: active site mutations define an isoform-specific network for allosteric signaling by cAMP. J Biol Chem 279: 7029-7036.

12. Gervasi N, Hepp R, Tricoire L, Zhang J, Lambolez B, Paupardin-Tritsch D, Vincent P (2007) Dynamics of protein kinase A signaling at the membrane, in the cytosol, and in the nucleus of neurons in mouse brain slices. J Neurosci 27: 2744-2750.

13. Endo S, Zhou X, Connor J, Wang B, Shenolikar S (1996) Multiple structural elements define the specificity of recombinant human inhibitor-1 as a protein phosphatase-1 inhibitor. Biochemistry 35: 5220-5228.

14. Bibb JA, Nishi A, O'Callaghan JP, Ule J, Lan M, Snyder GL, Horiuchi A, Saito T, Hisanaga S, Czernik AJ, Nairn AC, Greengard P (2001) Phosphorylation of protein phosphatase inhibitor-1 by Cdk5. J Biol Chem 276: 14490-14497.

15. Dupont G, Houart G, De KP (2003) Sensitivity of CaM kinase II to the frequency of Ca2+ oscillations: a simple model. Cell Calcium 34: 485-497.

16. De Koninck P, Schulman H (1998) Sensitivity of CaM kinase II to the frequency of Ca2+ oscillations. Science 279: 227-230.

17. Waxham MN, Tsai AL, Putkey JA (1998) A mechanism for calmodulin (CaM) trapping by CaM-kinase II defined by a family of CaM-binding peptides. J Biol Chem 273: 17579-17584.

18. Ajay SM, Bhalla US (2004) A role for ERKII in synaptic pattern selectivity on the time-scale of minutes. Eur J Neurosci 20: 2671-2680.

19. Hanson PI, Schulman H (1992) Neuronal Ca2+/calmodulin-dependent protein kinases. Annu Rev Biochem 61: 559-601.

20. Lee SJ, Escobedo-Lozoya Y, Szatmari EM, Yasuda R (2009) Activation of CaMKII in single dendritic spines during long-term potentiation. Nature 458: 299-304.
